# Application of Parameter Optimization Methods Based on Kalman Formula to the Soil—Crop System Model

**DOI:** 10.3390/ijerph20054567

**Published:** 2023-03-04

**Authors:** Qinghua Guo, Wenliang Wu

**Affiliations:** Beijing Key Laboratory of Biodiversity and Organic Farming, College of Resources and Environmental Sciences, China Agricultural University, Beijing 100193, China

**Keywords:** data assimilation, Bayesian calibration, WHCNS, parameter multimodal distribution, sampling efficiency

## Abstract

Soil–crop system models are effective tools for optimizing water and nitrogen application schemes, saving resources and protecting the environment. To guarantee model prediction accuracy, we must apply parameter optimization methods for model calibration. The performance of two different parameter optimization methods based on the Kalman formula are evaluated for a parameter identification of the soil Water Heat Carbon Nitrogen Simulator (WHCNS) model using mean bias error (ME), root-mean-square error (RMSE) and an index of agreement (IA). One is the iterative local updating ensemble smoother (ILUES), and the other is the DiffeRential Evolution Adaptive Metropolis with Kalman-inspired proposal distribution (DREAMkzs). Our main results are as follows: (1) Both ILUES and DREAMkzs algorithms performed well in model parameter calibration with the RMSE_Maximum a posteriori (RMSE_MAP) values were 0.0255 and 0.0253, respectively; (2) ILUES significantly accelerated the process to the reference values in the artificial case, while outperforming in the calibration of multimodal parameter distribution in the practical case; and (3) the DREAMkzs algorithm considerably accelerated the burn-in process compared with the original algorithm without Kalman-formula-based sampling for parameter optimization of the WHCNS model. In conclusion, ILUES and DREAMkzs can be applied to a parameter identification of the WHCNS model for more accurate prediction results and faster simulation efficiency, contributing to the popularization of the model.

## 1. Introduction

Unreasonable water and nitrogen management practices in intensive agricultural systems have caused serious resource waste and environmental pollution [1]. Field experiments and numerical simulations are the main approaches to optimize water and nitrogen application schemes for the construction of high-yield and low-pollution cropping systems [2,3,4]. Due to the long time consumption and high cost of field experiments, numerical models are crucial tools for analyzing how soil–crop systems respond to field management schemes. There are many relevant soil–crop system models, such as RZWQM [5], WNMM [6], SWAT [7] and SPWS [8]. DRAINMOD is often combined with other models, such as ADAPT, RZWQM2 and DSSAT [9,10,11,12]. There are also many other soil–crop models, such as HERMES [13], CHANI-EPIC [14], DAYCENT [15] and NLEAP-GIS [16]. DNDC can simulate the processes responsible for the production, consumption and transport of nitrous oxide [17]. The soil Water Heat Carbon Nitrogen Simulator (WHCNS) model can be applicable to studies of water and nitrogen management under the complex conditions of intensive cropping systems in North China [18].

However, parameters must be calibrated and validated before the application of soil–crop system models to ensure prediction accuracy [19,20,21,22]. There are several cases for exploring the calibration of system models through combining parameter optimization methods with state variable observations [23,24]. Quantum-behaved Particle Swarm Optimization (QPSO) was used for the parameter calibration of the RZWQM2 model [21,25]. The Bayesian method was also useful as a method for quantifying uncertainty [26]. The RZWQM2 model was calibrated by the generalized likelihood uncertainty estimation (GLUE) method, suggesting the application of likelihood function with probability theory [27]. GLUE and Markov Chain Monte Carlo (MCMC) were compared for the parameter identification of the APSIM-Sugar model [28,29]. The DREAM algorithm was introduced for the parameter optimization of the STICS model and DayCent agroecosystem model [30,31]. However, the applicability of current popular parameter inversion methods to the data assimilation of soil–crop system models still needs further exploration.

In recent years, data assimilation methods have rapidly developed and are widely used in various fields. Ensemble Kalman filter (EnKF) can be considered for parameter optimization in nonlinear problems [32]. As its extension, ensemble smoother (ES) is a more efficient data assimilation method, which avoids updating model states and parameters simultaneously [33]. Surrogate-based iterative ES can also increase computation efficiency by constructing surrogates using the Gaussian process [34], polynomial chaos and so on. ES can also be coupled with other methods to solve data assimilation in non-Gaussian-distributed conductivity fields [35,36]. However, neither EnKF nor ES perform well when the model parameter distributions are multimodal. An iterative local updating ensemble smoother (ILUES) was proposed to explore the possible multimodal distributions of the hydrologic model’s parameters [37]. Currently, MCMC algorithms are relatively popular for exploring multimodal parameter distributions, even though they are proposed based on the Gaussian assumption. However, the computational cost is prohibitive, especially for a high-dimensional model. Implementing the Kalman formula for the DREAM algorithm’s proposal distribution improves sampling efficiency [38]. The introduction of the above-mentioned newly proposed parameter optimization methods for the data assimilation of soil–crop systems can improve the model’s prediction accuracy and promote its wide application.

We hypothesize that the ILUES and DREAMkzs data assimilation methods will increase the WHCNS model’s simulation accuracy and calibration efficiency. Our study objectives are to (i) compare the efficiency of exploring parameter space between ILUES and ESMDA using synthetic soil water content derived by the WHCNS model, (ii) evaluate the feasibility of exploring the multimodal distribution of the ILUES algorithm’s model parameters, and (iii) compare the WHCNS model’s sampling efficiency for DREAMkzs and DREAMzs using soil water content observations.

## 2. Materials and Methods

### 2.1. Introduction of Data Assimilation Methods

#### 2.1.1. Basic Theory of Iterative Local Updating Ensemble Smoother

The general form of ES is shown as follows [39]:

(1) Δm= G (Δy),

where ∆**m** is the update vector, ∆**y** is the innovation vector, and G is a mapping from ∆**y** to ∆**m**, which is defined by the Kalman gain matrix **K**.

The essence of the ILUES method is to update local ensembles of each sample instead of the global ensembles. The local ensembles for a realization $m_{j}^{f}$ can be derived through the following equation [40]:

(2) $$J\;(m)=a*J_{1}\;(m)\;/J_{1}^{max}+b*J_{2}\;(m)\;/J_{2}^{max},$$

where $J_{1}$ (**m**) = ${\left[d-f\left(m\right)\right]}^{T}C_{D}^{-1}\left[d-f\left(m\right)\right]$ represents the distance between the simulation results f(m) and the measurements **d**; $J_{2}$ (**m**) = ${\left[m-m_{j}^{f}\right]}^{T}C_{MM}^{-1}\left[m-m_{j}^{f}\right]$ represents the distance between the realization $m_{j}^{f}$ and parameters **m**, and $C_{MM}$ represents the model parameter auto-covariance matrix; and the maximum values of $J_{1}$ (**m**) and $J_{1}$ (**m**) are defined as $J_{1}^{max}$ and $J_{2}$ (**m**) is $J_{2}^{max}$, respectively. $M_{j,L}^{f}=[m_{j,1}^{f},\ldots ,m_{j,N_{L}}^{f}$] containing the realizations with $N_{L}=\alpha N_{e}$ and smallest *J* is defined as the local ensemble for $m_{j}^{f}$. Then, ILUES updates $M_{j,L}^{f}$ to $M_{j,L}^{a}$ using the following equation:

(3) $$m_{j,l}^{a}=m_{j,l}^{f}+C_{MD}^{L,f}(C_{DD}^{L,f}+C_{D}{)}^{-1}[d_{j}-f\left(m_{j,l}^{f}\right)],$$

where $C_{MD}^{L,f}$ represents the cross-covariance matrix between the model parameters $M_{j,L}^{f}$ and the corresponding simulation results $D_{j,L}^{f}$, and $C_{DD}^{L,f}$ represents the $D_{j,L}^{f}$ auto-covariance matrix, where *l* = 1, …, $N_{L}$. Then, the possible multimodel distributions of model parameters may be achieved by choosing the updated realizations $m_{j}^{a}$ randomly from the updated local ensemble $M_{j,L}^{a}$.

The multiple data assimilation (MDA) scheme was applied as a supplementary for the model parameter update to achieve better optimization results for nonlinear problems [41,42]. The iterative form of an ES, which is defined as an ESMDA, assimilates the observations multiple times. The factor $\alpha_{i}$ should be applied to inflate the measurement error for obtaining the reasonable results in iteration i. The factor should obey $\overset{N}{\underset{i=1}{\sum }}1/\alpha_{i}{}^{2}$ = 1, where N is the total assimilation time.

#### 2.1.2. Theory of the DREAMkzs Algorithm

The formula of the Kalman-inspired proposal distribution, which accelerates the convergence of the chain to the posterior distribution, is shown as follows:

(4) $$\theta_{p}=\theta_{\left(t-1\right)}+Kr_{\left(t-1\right)}+K\epsilon_{\left(t-1\right)},$$

where θ is a vector of model parameters; $r_{\left(t-1\right)}$ represents the residual vector of parameters $\theta_{\left(t-1\right)}$, and $\epsilon_{\left(t-1\right)}$ denotes a vector of random draw from the distribution of measurement errors ($\epsilon_{\left(t-1\right)}\sim N\left(0,R\right)$, with **R** as the covariance matrix of measurement errors. The Kalman gain **K** is defined as:

(5) $$K=C_{\theta d}(C_{dd}+R{)}^{-1},$$

where $C_{\theta d}$ signifies the cross-covariance matrix of model parameters and simulation results, and $C_{dd}$ denotes the covariance matrix of model simulation results.

The candidate can be derived from the samples in the archive, through the mixture of parallel direction, snooker and Kalman trial moves. However, the Kalman-inspired proposal distribution introduces asymmetry even though it can shorten the burn-in processes. The approaches that can remedy this defect at present will considerably deteriorate the sampling efficiency. To avoid this disadvantage, the Kalman-inspired proposal distribution is only applied to the first $T_{k}$ steps of the Markov chain, after which the parallel direction and snooker proposal distributions are used for sampling.

### 2.2. Introduction of Forward Model Information

#### 2.2.1. WHCNS Model

The WHCNS, which has a detailed introduction in the literature [43], was selected as the forward soil–crop system model for our simulation. The depth of the soil profile was 180 cm, assuming that nitrogen moved beyond this depth could not be utilized by the crop. In total, 32 uncertain hydraulic parameters are listed in Table 1. The soil profile was divided into eight layers with four parameters for each layer. Considering the 8 crop parameters and 5 nitrogen transformation parameters shown in Table 2, there were 45 parameters that needed to be calibrated in all. The prior distributions of the parameters were assumed to be uniform distributions, and the ranges are listed in Table 1 and Table 2.

Mean bias error (ME), root mean square error (RMSE) and index of agreement (IA) are three different statistics applied for the evaluation of the model performance. The corresponding equations for them are as follows:

(6) $$\mathrm{ME}=\;\sum_{i=1}^{n}\frac{\mathrm{Si}-\mathrm{Oi}}{n},$$

(7) $$\mathrm{RMSE}=\;\sqrt{\frac{\sum_{i=1}^{n}\;\left(\mathrm{Si}-\mathrm{Oi}\right){\;}^{2}}{n},}$$

(8) $$\mathrm{IA}=1-\frac{\sum_{i=1}^{n}\;\left(\mathrm{Si}-\mathrm{Oi}\right){\;}^{2}}{\sum_{i=1}^{n}\left(\left|\mathrm{Si}-\bar{O}\right|+\left|\mathrm{Oi}-\bar{O}\right|\right){\;}^{2\;}},$$

where *n* is the number of observations, Si is the simulated value, Oi is the measured value, and $\bar{O}$ is the mean of the measured values. The RMSE represents the average difference between the simulations and the observations. The range of IA is limited to 0–1, and a better model performance is indicated if the value is closer to 1.

#### 2.2.2. Description of Field Experiment Conditions

The study area was located within Alxa Left Banner, Inner Mongolia, China. The total potential evaporation is 20 times more than the average annual precipitation, which reaches 116 mm/year. The oasis cropping system is single crop growth during middle April to early October, which is dominated by spring maize. Irrigation is typically applied as flood irrigation and depends on groundwater, ranging from 40 to 70 m in depth. Typical N fertilizer application rates are about 280–350 kg N ${\mathrm{ha}}^{-1}$ [44]. The field experiment was undertaken at the experimental site over two spring maize growth periods. Four treatments were replicated three times in the 20 m × 20 m experimental plots each year. Maize was planted on April 12 and harvested on October 18. Volumetric soil water content was measured from 16 points of the soil profile by taking a value every 10 cm from a depth of 20 cm to 170 cm on 17 days (the 41st, 52nd, 54th, 63rd, 70th, 74th, 82nd, 91st, 95th, 104th, 109th, 115th, 124th, 135th, 146th, 154th and 160th day after sowing), using time domain reflectometry (TDR) probes. Detailed information on water and N management practices are presented in Table 3 [45]. Four irrigation-fertilizer treatments, namely I_std_N_std_ (W_1_N_1_), I_std_N_csv_ (W_1_N_2_), I_csv_N_std_ (W_2_N_1_) and I_csv_N_csv_ (W_2_N_2_), can be derived by combining two irrigation treatments with two N-fertilization applications.

## 3. Results

We demonstrate the performance of the ILUES compared with the ESMDA through both synthetic and practical cases, which are mentioned in Section 3.1.1 and Section 3.1.2, respectively. Then, the applicability of the DREAMkzs algorithm is evaluated in Section 3.2. The parameters that need to be optimized include 32 soil hydraulic parameters for 8 layers of the soil profile, 5 nitrogen transformation parameters and 8 crop parameters, which are contained in Table 1 and Table 2. Uniform prior parameter distributions are assumed based on the ranges listed in Table 1 and Table 2.

### 3.1. Comparison of ILUES and ESMDA

#### 3.1.1. Synthetic Case

**Figure 1:**
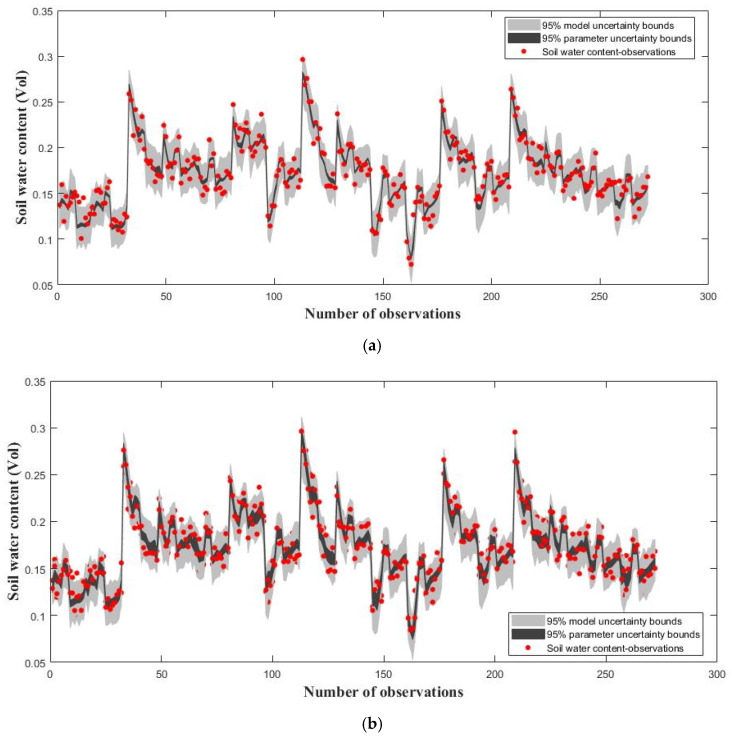
Plots of simulated and reference soil water content. 95% simulation uncertainty intervals due to parameter (dark region) and total uncertainty (light gray). The reference soil water content values are indicated with red points. (**a**) iterative local updating ensemble smoother (ILUES); (**b**) ensemble smoother multiple data assimilation (ESMDA).

To demonstrate the performance of the ILUES algorithm, a synthetic case is discussed in this section. The reference soil water content values indicated with red points in Figure 1a,b are derived through the following steps: First, randomly sample the reference parameter values from the corresponding prior uniform distributions consistent with the ranges listed in Table 1 and Table 2; second, generate the reference output values by collecting soil water content at 16 different points of the soil profile on the 17 days mentioned in Section 2.2.2, which are derived by running the forward WHCNS model; and third, disturb the simulated soil water content values using the assumed measurement error $\varepsilon \sim N\;\left(0,\;0.005^{2}\right).$ The ILUES and MDAES algorithms are executed with the number of iterations as 3 and an ensemble size of 500. Figure 1a shows the fitting results of the simulated soil water content and the corresponding reference values derived by the ILUES algorithm with the RMSE_Maximum a posteriori (RMSE_MAP) is equal to 0.0104, while Figure 1b shows similar results derived by the MDAES algorithm with RMSE_MAP = 0.0093. Then, we come to a preliminary conclusion that the ILUES algorithm is able to obtain a satisfactory fitting effect equivalent to ESMDA.

**Figure 2:**
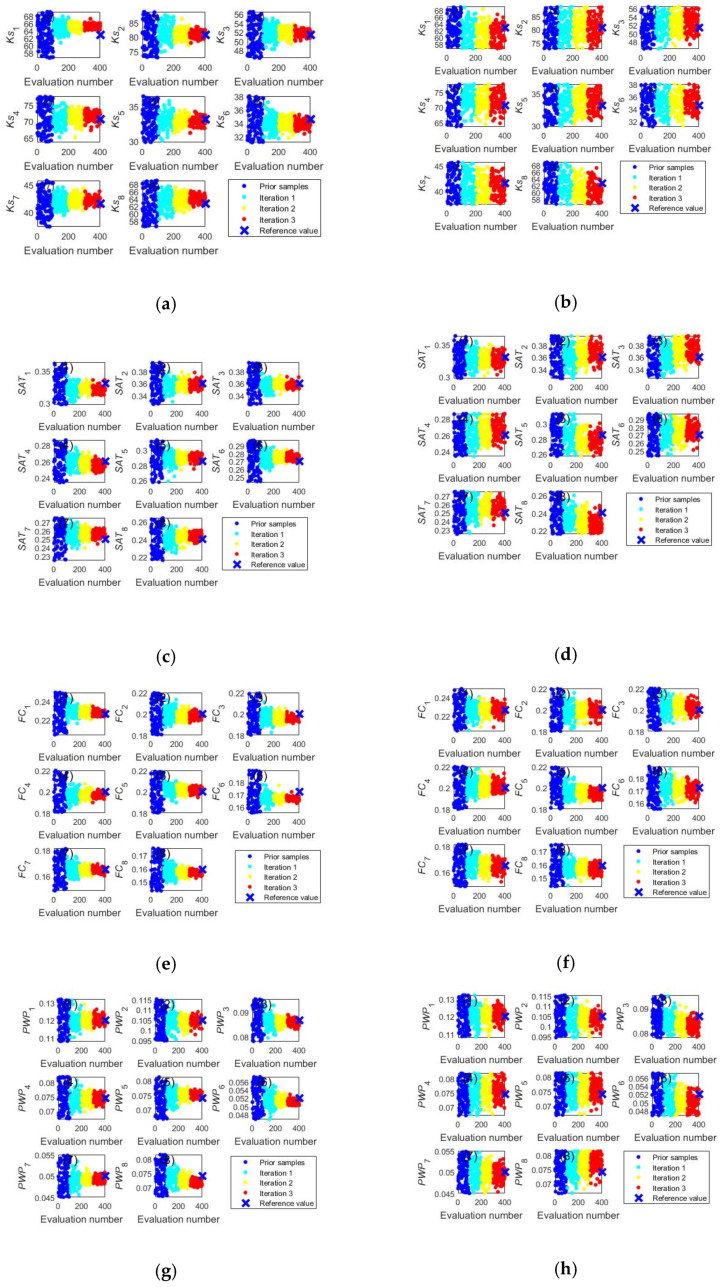
Trace plots of soil hydraulic parameters obtained by the ILUES (**left column**) and ESMDA (**right column**) algorithms. The blue cross symbol at the right side of each figure signifies the referred value of each parameter. (**a**) Saturated hydraulic conductivity derived by ILUES; (**b**) Saturated hydraulic conductivity derived by ESMDA; (**c**) Saturated soil water content derived by ILUES; (**d**) Saturated soil water content derived by ESMDA; (**e**) Field capacity derived by ILUES; (**f**) Field capacity derived by ESMDA; (**g**) Wilting point derived by ILUES; (**h**) Wilting point derived by ESMDA.

**Figure 3:**
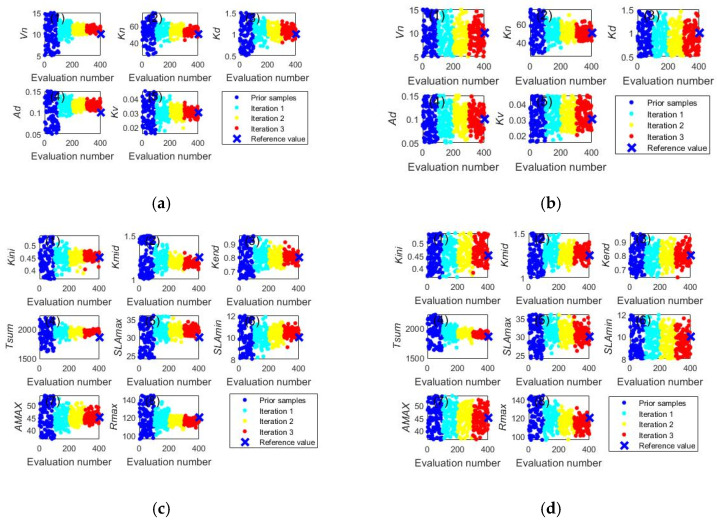
Trace plots of soil nitrogen transformation parameters and crop parameters obtained by the ILUES (**left column**) and ESMDA (**right column**) algorithms. The blue cross symbol at the right side of each figure signifies the referred value of each parameter. (**a**) Soil nitrogen transformation parameters derived by ILUES; (**b**) Soil nitrogen transformation parameters derived by ESMDA; (**c**) Crop parameters derived by ILUES; (**d**) Crop parameters derived by ESMDA.

Figure 1 shows that acceptable simulation accuracy can be obtained by both the ILUES and ESMDA algorithms after three iterations. To further demonstrate the proposed algorithm’s parameter estimation capabilities, the trace plots of model parameters calibrated by the two methods are compared in Figure 2 and Figure 3. In Figure 2, the ILUES algorithm’s sampling efficiency for all soil hydraulic parameters is higher than that of the ESMDA algorithm because its sampling trace derived converged to the corresponding reference values faster. Figure 3 shows a similar performance, except for T_sum_. This result indicates that the ILUES method’s ability to explore the parameter space is superior to the ESMDA method, as noted in the literature [40,41].

#### 3.1.2. Practical Case

**Figure 4:**
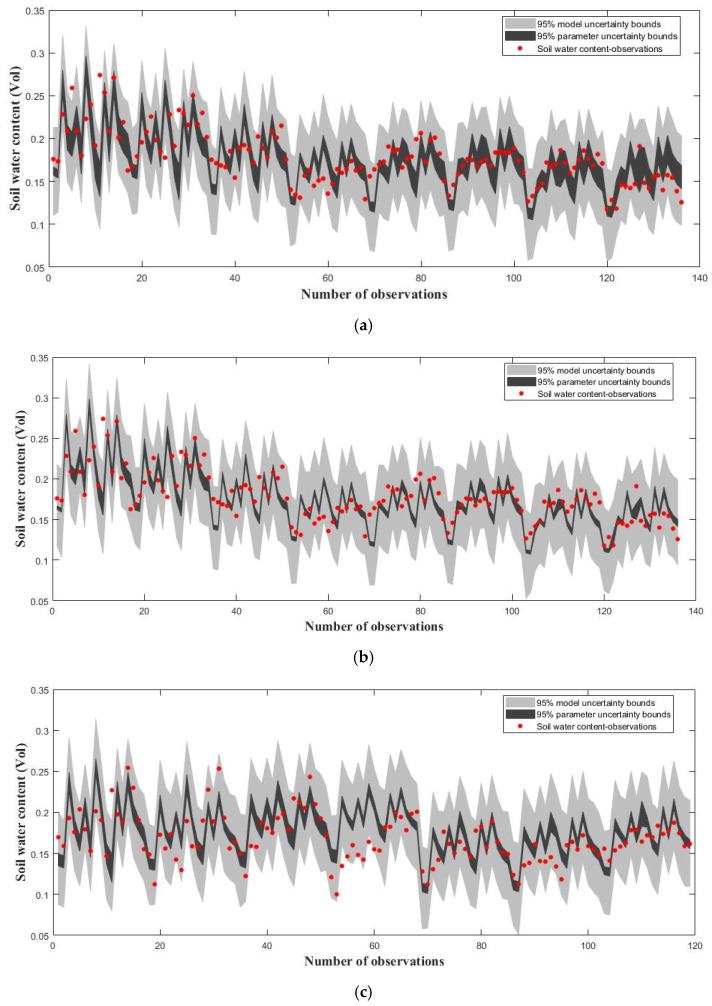

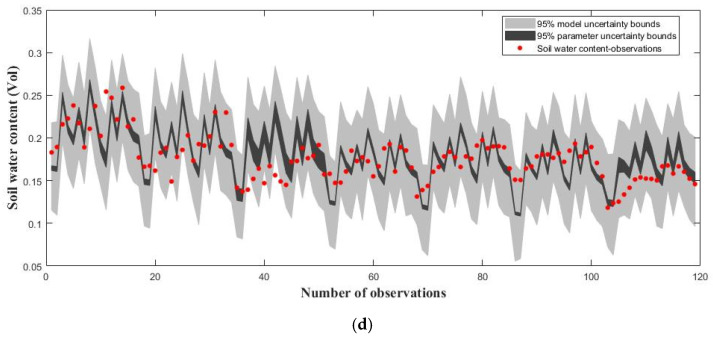
Plots of simulated and reference soil water content. 95% simulation uncertainty intervals due to parameter (dark region) and total uncertainty (light gray). The measured soil water content values are indicated with red points. (**a**) ILUES-calibration; (**b**) ESMDA-calibration; (**c**) ILUES-validation; (**d**) ESMDA-validation.

To further demonstrate the ILUES algorithm’s performance compared with the ESMDA method, the soil hydraulic, crop and nitrogen parameters of the WHCNS model are simultaneously identified through the practical W_2_N_1_ case mentioned in Table 3. The red points shown in Figure 4a,b indicate volumetric soil water content measured from depths of 20 cm, 40 cm, 60 cm, 80 cm, 100 cm, 120 cm, 140 cm and 160 cm; furthermore, those displayed in Figure 4c,d indicate volumetric soil water content measured from depths of 30 cm, 50 cm, 70 cm, 90 cm, 110 cm, 130 cm and 170 cm. Volumetric soil water content at each selected depth of soil profile are measured at 17 days, which is mentioned in Section 2.2.2. That is to say, there are 136 observations at 8 layers for 17 days that need to be calibrated, and 119 measurement values at 7 layers for 17 days are used to validate the calibrated model’s capacity for accurate prediction. Figure 4a,b are the calibration results of the ESMDA and ILUES algorithms, and the RMSE_MAP values for both are 0.0255, meaning both methods have a similar data assimilation capability. Figure 4c,d show validation results for the two different inverse methods with RMSE_MAPs, which are 0.0265 and 0.0269, respectively, indicating that the ILUES algorithm can derive reasonable results without overfitting. Therefore, both methods can reliably optimize the forward model system for accurate predictions.

**Figure 5:**
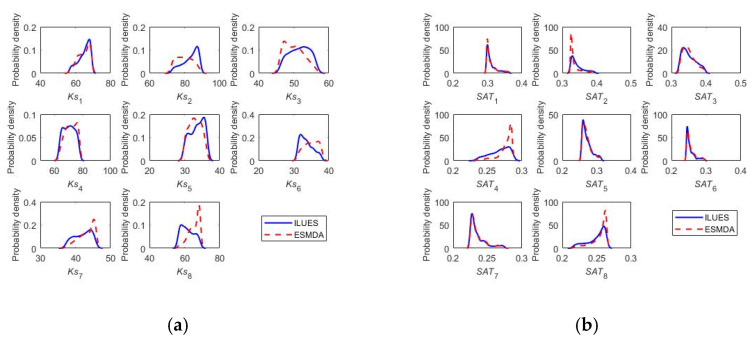

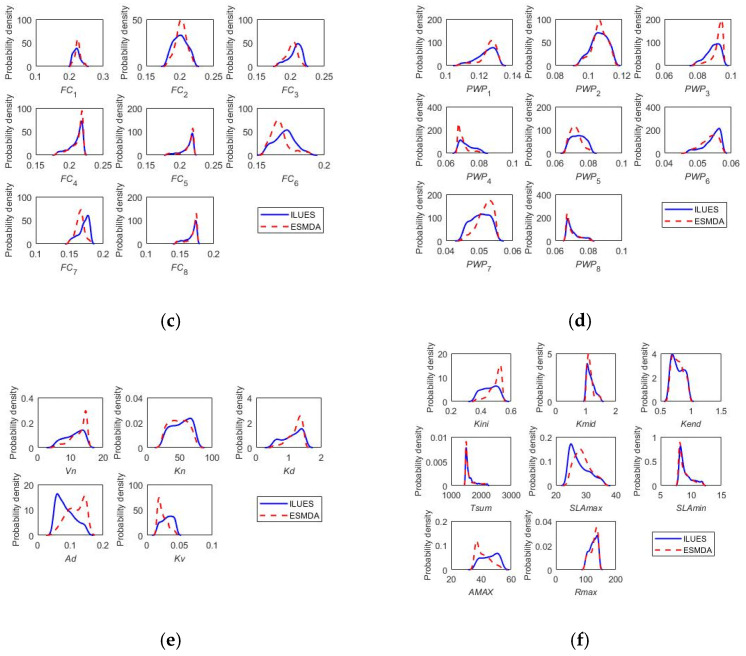
Posterior marginal distributions of model parameters estimated by ILUES (blue lines) and ESMDA (red dashed lines), respectively. (**a**) Ks; (**b**) Saturated soil water content; (**c**) Field capacity; (**d**) Wilting point; (**e**) Nitrogen transformation parameters; (**f**) Crop parameters, for W_2_N_1_ listed in Table 3.

Figure 5 presents the posterior distributions of model parameters obtained by the two inverse algorithms. Overall, reliable and robust estimations of the soil and crop parameters can be derived by the two ES-based methods. For Ks_1_, Ks_4_, SAT_1_, SAT_3_, SAT_5_, SAT_6_, SAT_7_, FC_5_, FC_8_, PWP_8_, T_sum_ and SLA_max_, the posterior distributions derived by the ILUES method almost coincided with the corresponding results obtained by ESMDA. For Ks_5_, Ks_7_, Ks_8_, FC_6,_ FC_7_, PWP_3_, PWP_4_, PWP_6_, K_ini_, AMAX and five nitrogen transformation parameters, ILUES extracted the multimodal posterior distributions of parameters more obviously than ESMDA. Therefore, we conclude that, for a practical case with unknown posterior parameter distributions, ILUES is superior in exploring the multimodal distributions compared with ESMDA, which is consistent with the literature [38].

### 3.2. Comparison of DREAMkzs and DREAMzs

#### 3.2.1. Model Performance Evaluation

**Figure 6:**
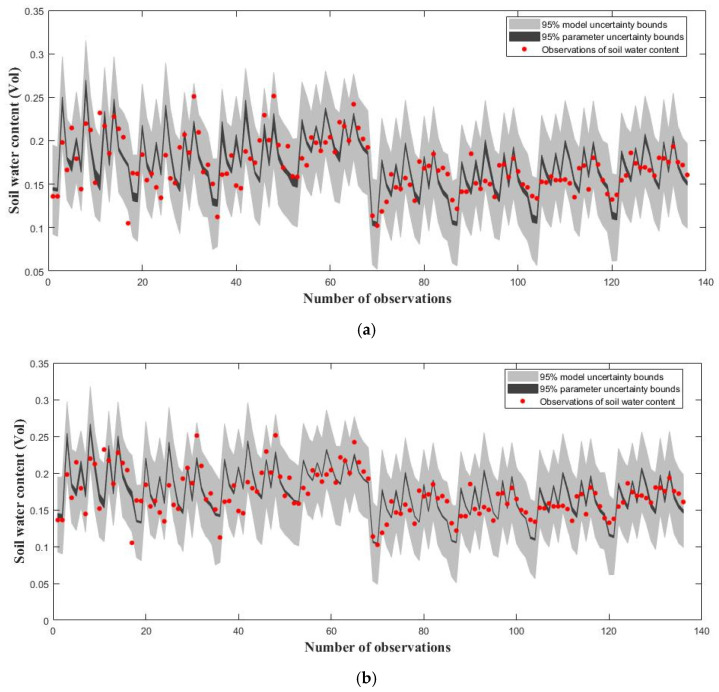
Plots of simulated and reference soil water content. 95% simulation uncertainty intervals due to parameter (dark region) and total uncertainty (light gray). The measured soil water content values are indicated with red points. (**a**) DiffeRential Evolution Adaptive Metropolis with Kalman-inspired proposal distribution (DREAMkzs); (**b**) DiffeRential Evolution Adaptive Metropolis (DREAMzs).

Figure 6 shows evaluations of the DREAMzs and DREAMkzs algorithms’ performances by applying the WHCNS model’s parameter calibration based on the practical W_1_N_1_ case mentioned in Table 3. We take P_p_ = 0.7, P_s_ = 0.1, P_k_ = 0.2 and T_k_ = 0.2T, where P_p_, P_s_ and P_k_ represent the selection probabilities of the parallel direction, snooker and Kalman-inspired proposal distributions, respectively, and T denotes the total number of model evaluations. Both MCMC algorithms were run with the number of chains N set to 3 and the number of generations in each chain T set to 1000. The red points shown in Figure 6a,b indicate the volumetric soil water content measured from depths of 20 cm, 40 cm, 60 cm, 80 cm, 100 cm, 120 cm, 140 cm and 160 cm. Volumetric soil water content at each selected soil profile depth is measured at 17 days, which is mentioned in Section 2.2.2. In total, 136 observations at 8 layers for 17 days need to be calibrated. Figure 6a,b show that the MCMC methods, including both DREAMkzs and DREAMzs, performed well in improving the level of fit between the observed states and simulated results, with RMSE_MAP values of 0.0253 and 0.0255, respectively. This preliminarily proved that the recommended DREAMkzs algorithm is feasible for accurately estimating practical observations.

#### 3.2.2. Comparison of Simulation Statistics

**Figure 7:**
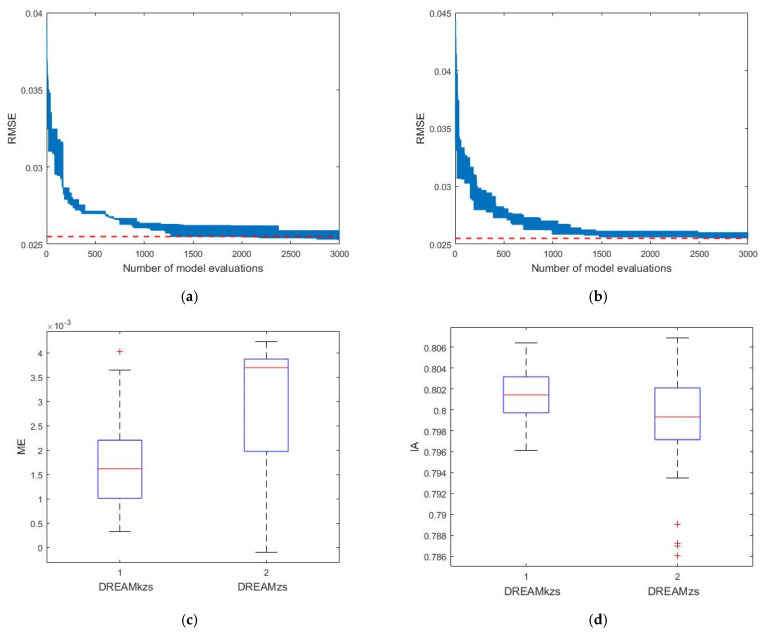
Statistics analysis of calibration results. (**a**) Evolution of RMSE derived by DREAMkzs. (**b**) Evolution of RMSE derived by DREAMzs. (**c**) Boxplots of ME. (**d**) Boxplots of IA.

Figure 7a,b display the evolution of the RMSE between the simulated and observed soil water content derived by DREAMkzs and DREAMzs, respectively. The horizontal red dashed line depicts the reference value of 0.0255. DREAMkzs significantly accelerates the optimization process compared with DREAMzs. Figure 7c,d show the distributions of ME and IA obtained by Equations (6) and (8), respectively. The results indicate that the simulated soil water content derived by both algorithms agreed well with the practical observations. Moreover, DREAMkzs improves the sampling efficiency considerably more than DREAMzs. However, the number of generations is small because the original parameter ranges are determined based on the LM algorithm’s optimized results. More generations may be required if there are no preliminary optimization results.

## 4. Discussion

We found that the proposed parameter optimization methods can explore parameter sampling spaces and considerably improve sampling efficiency, increasing the WHCNS model’s prediction accuracy. Reliable simulation results can be derived by the calibrated models, which popularize the WHCNS model. In addition, models with high prediction accuracy can be applied to analyze the effect of climate, types of plants and field management on soil water movement, crop growth and soil nitrogen transport, contributing to the development of field management practices that not only save resources but also decrease the environmental pollution induced by excessive water and fertilizer application.

Obvious differences exist between the two Kalman-formula-based parameter optimization methods mentioned above, even though both of them can identify the model with acceptable accuracy. First, the Kalman formula plays different roles in the two proposed parameter optimization algorithms. The Kalman formula is utilized to update the parameter ensemble, while the Kalman-inspired proposal distribution is just applied to sample a parameter candidate in the DREAMkzs algorithm. Second, the influence degree of the Kalman formula on inversion results is different. In ILUES, the Kalman gain exists throughout the whole parameter inversion process [40]. However, the Kalman-inspired proposal distribution is only used in the first several steps to avoid introducing asymmetry in the sampled candidate states [46]. In addition, parameter calibration results that enable a good fitness to the observed data can make a satisfying prediction under the same treatment. However, it does not guarantee comparable estimations accuracy for other types of field management practices.

ILUES and DREAMkzs are newly proposed parameter inversion methods that have not been previously applied in the data assimilation of soil–crop system models. However, soil water content was the single-state variable used for the models’ parameter calibrations, and improvements can be made by considering other state variables, such as soil nitrate concentration, crop N uptake and yield [30]. Meanwhile, we consider the soil hydraulic, soil nitrogen transformation and crop parameters simultaneously, which induced a high dimension. Global sensitivity analysis can be adopted to reduce parameter dimensions, as in [47]. In addition, the effects of applying the two proposed methods to high nonlinear models need to be further verified. Meanwhile, an increase in the parameter dimension can reduce the sampling efficiency. Therefore, more attention should be paid to further improving the parameter optimization performance of soil–crop system models.

## 5. Conclusions

Both ILUES and DREAMkzs are parameter optimization methods based on the Kalman formula. They can be successfully applied to a data assimilation of the WHCNS model, even though the Kalman formula produces a different effect for them. Our main conclusions are as follows: (1) The models calibrated using both ESMDA and ILUES algorithms under the condition of W_2_N_1_ are promisingly robust. The parameter distribution approaches the reference value faster under the artificial situation. Moreover, the performance of ILUES in modeling multimodal parameter distributions under a practical situation is superior to that of ESMDA under the same number of model evaluations condition. (2) DREAMkzs was successful in considerably accelerating the burn-in procedure compared with the DREAMzs with a comparative soil water content fitting accuracy under the same water and nitrate management practices.

Accurate model prediction is conducive to the formulation of field management measures for energy conservation and high yield. Furthermore, reducing input cost and increasing yield can raise farmers’ incomes. The applicability of the latest parameter inversion methods in other fields to the WHCHS model can also be evaluated. At the same time, the two data assimilation methods mentioned in this paper can also be applied to other soil–crop system models. In addition, deep learning is currently popular and not limited by Gaussian hypothesis; therefore, it can be considered for introduction into the parameter inversion of a soil–crop system model, whether in whole or part.

## Figures and Tables

**Table 1:** Soil hydraulic parameters selected for calibration.

Name		Saturated Hydraulic Conductivity	Saturated Soil Water Content	Field Capacity	Wilting Point
Symbol		Ks	SAT	FC	PWP
Unit		cm/d	cm^3^/cm^3^	cm^3^/cm^3^	cm^3^/cm^3^
Interval	1	[56.59–69.17]	[0.30–0.36]	[0.20–0.25]	[0.11–0.13]
	2	[72.58–88.70]	[0.32–0.40]	[0.18–0.22]	[0.09–0.12]
	3	[46.22–56.50]	[0.32–0.40]	[0.18–0.22]	[0.08–0.10]
	4	[63.50–77.62]	[0.23–0.29]	[0.18–0.22]	[0.06–0.08]
	5	[29.81–36.43]	[0.26–0.31]	[0.16–0.19]	[0.06–0.08]
	6	[31.10–38.02]	[0.24–0.30]	[0.15–0.18]	[0.04–0.06]
	7	[37.37–45.67]	[0.23–0.28]	[0.14–0.18]	[0.04–0.06]
	8	[56.38–68.90]	[0.22–0.26]	[0.10–0.13]	[0.06–0.08]

**Table 2:** Nitrogen transformation and crop parameters selected for calibration.

Types of Parameters	Description	Symbol	Unit	Interval
Nitrogen transformation parameters	Maximum nitrification rate	Vn	g/($m^{3}\cdot d$)	[5–15]
	Nitrification semi saturation constant	Kn	g/$m^{3}$	[25–75]
	Denitrification ratio constant	Kd	-	[0.50–1.50]
	Empirical constant of denitrification	Ad	-	[0.05–0.15]
	First order kinetic constant of ammonia volatilization	Kv	$d^{-1}$	[0.015–0.045]
Crop parameters	Initial crop coefficient	$K_{ini}$	-	[0.36–0.54]
	Medium term crop coefficient	$K_{mid}$	-	[1–1.5]
	Late crop coefficient	$K_{end}$	-	[0.64–0.96]
	Accumulated temperature from emergence to maturity	$T_{sum}$	°C	[1480–2220]
	Maximum specific leaf area	${\mathrm{SLA}}_{\max}$	$m^{2}/\mathrm{kg}$	[24–36]
	Minimum specific leaf area	${\mathrm{SLA}}_{\min}$	$m^{2}/\mathrm{kg}$	[8–12]
	Maximum assimilation rate	AMAX	kg/(${\mathrm{hm}}^{2}\cdot h$)	[36–54]
	Maximum root depth	$R_{max}$	cm	[96–144]

**Table 3:** Irrigation and fertilizer schemes of spring maize.

Treatment	Date of Scheduled Irrigation/Fertilizer Application						
	2008	3 June	21 June	13 July	4 August	29 August	Seasonal Total
	2009	1 June	22 June	13 July	1 August	23 August	
Irrigation (mm)							
Istd		150	150	150	150	150	750
Icsv		105	105	120	120	120	570
N fertilization (kg Urea-N/ha)							
Nstd		138					138
Ncsv		92					92

## Data Availability

Not applicable.
